# Machine learning and network analysis with focus on the biofilm in *Staphylococcus aureus*

**DOI:** 10.1016/j.csbj.2024.11.011

**Published:** 2024-11-10

**Authors:** Zhiyuan Zhang, Guozhong Chen, Wajid Hussain, Yuanyuan Pan, Zhu Yang, Yin Liu, Erguang Li

**Affiliations:** a Jiangsu Key Laboratory of Molecular Medicine, Medical School, Nanjing University, Nanjing, Jiangsu 210093, China; b Department of Medical Information Engineering, School of Medical Information, Wannan Medical College, Wuhu 241000, China; c State Key Laboratory of Microbial Technology, Shandong University, Qingdao, Shandong, China; d Advanced Biomaterials and Tissue Engineering Center, College of Life Science and Technology, Huazhong University of Science and Technology, Wuhan 430074, China; e Department of Medical Microbiology and Immunology, Wannan Medical College, Wuhu, Anhui, China

**Keywords:** Staphylococcus aureus, Biofilm, Machine learning, WGCNA, Databse

## Abstract

Research on biofilm formation in *Staphylococcus aureus* has greatly benefited from the generation of high-throughput sequencing data to drive molecular analysis. The accumulation of high-throughput sequencing data, particularly transcriptomic data, offers a unique opportunity to unearth the network and constituent genes involved in biofilm formation using machine learning strategies and co-expression analysis. Herein, the available RNA sequencing data related to *Staphylococcus aureus* biofilm studies and identified influenced functional pathways and corresponding genes in the process of the transition of bacteria from planktonic to biofilm state by employing machine learning and differential expression analysis. Using weighted gene co-expression analysis and previously developed online prediction platform, important functional modules, potential biofilm-associated proteins, and subnetworks of the biofilm-formation pathway were uncovered. Additionally, several novel protein interactions within these functional modules were identified by constructing a protein-protein interaction (PPI) network. To make this data more straightforward for experimental biologists, an online database named SAdb was developed (http://sadb.biownmcli.info/), which integrates gene annotations, transcriptomics, and proteomics data. Thus, the current study will be of interest to researchers in the field of bacteriology, particularly those studying biofilms, which play a crucial role in bacterial growth, pathogenicity, and drug resistance.

## Graphical Abstract

**Figure:**
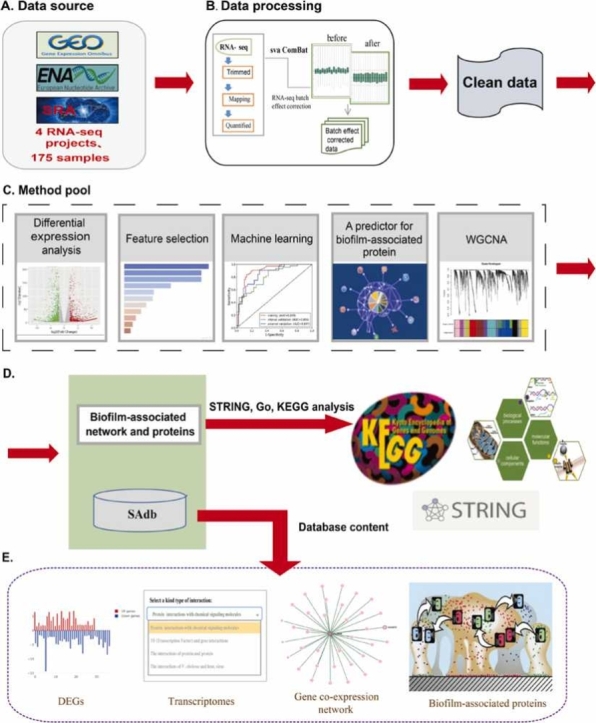

## 1 Introduction

*Staphylococcus aureus*, a ubiquitous pathogen, is implicated in a myriad of afflictions ranging from pneumonia and sepsis to infections of the wound and urinary tract [1]. The ability of this pathogen to form a bacterial biofilm poses a formidable challenge for medical intervention [2], [3]. Biofilms are extracellular polymeric substance (EPS) matrix composed of polysaccharides, proteins, extracellular DNA, and lipids. Biofilm-associated infections account for 65–80 % of all human microbial infections, leading to serious mortality and morbidity [4]. Biofilm-enriched bacteria exhibit approximately thousand-fold increased resistance to antimicrobial agents compared with their planktonic counterparts [5]. Thus, elucidating the biofilm development mechanism of *Staphylococcus aureus* is imperative to enhance its treatment efficacy and provide a theoretical framework for its prevention and management.

The primary components of bacterial biofilms include extracellular DNA (eDNA), polysaccharides, various proteins, and other bacterial secretions [6]. Among these, biofilm-associated proteins constitute a vital category that includes both the structural components of the biofilm and regulatory elements governing biofilm formation. Exploration and identification of these proteins are crucial for understanding the molecular intricacies of bacterial biofilm formation. Typically, these proteins are identified through experiments where, for instance, proteins such as CcpA, CodY, and GltS have been shown to influence the formation of biofilms in *Staphylococcus aureus* [7], [8]. Moreover, when compared to other bacteria like *Pseudomonas aeruginosa,* whose biofilm pathway documented in the KEGG database encompasses 110 proteins, and *Vibrio cholerae that* the corresponding biofilm network includes 75 proteins, the biofilm networks of *Staphylococcus aureus* have yet to be annotated in prevalent databases. This highlights a substantial gap in our knowledge and underscores the potential for groundbreaking research in this domain.

Despite advances in omics research, *Staphylococcus aureus* has greatly benefited from next-generation sequencing (NGS) technologies [9]. The power of meta-analysis has been amplified by the integration of high-quality bacterial NGS data, allowing for more robust conclusions from various experimental results. In particular, expression data lend themselves well to such integrative analysis, facilitating the categorization of genes into functional modules through co-expression metrics. In microbial research, methodologies such as weighted gene co-expression network analysis (WGCNA) have proven invaluable in delineating critical genetic interactions under the “guilt by association” principle [10]. Moreover, machine learning techniques continue to provide new biological insights by analyzing vast and varied datasets, particularly microarray and RNA sequencing data [11].

The RNA sequencing data from *Staphylococcus aureus* under biofilm phenotype were united and analyzed the data using differential expression analysis, machine learning, weighted correlation network analysis (WGCNA), and developed predictor (http://124.222.145.44/#!/score). Additionally, to facilitate easy access to available data, an online database named SAdb was developed, which also collects, organizes *Staphylococcus aureus* gene expression data, gene annotation information, and proteomic data, available publicly at http://sadb.biownmcli.info/. This may lead to the identification of important proteins and their interactions in the biofilm phenotype using a comprehensive analysis strategy.

## 2 Results

### 2.1 Analysis procedures

**Fig. 1:**
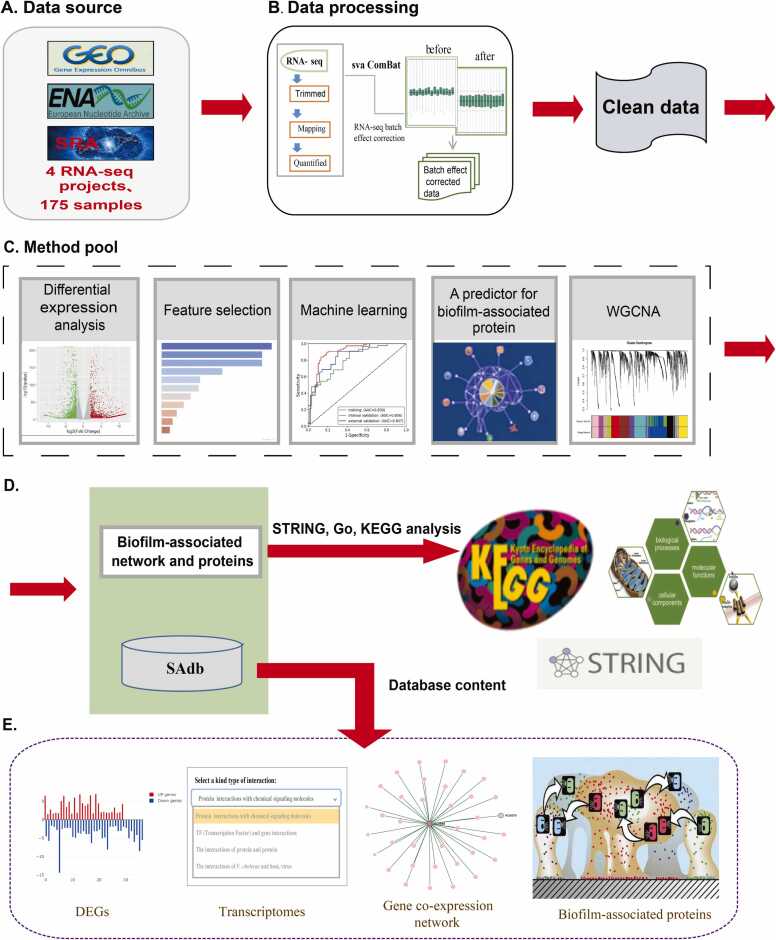
(A) Workflow of analysis. RNA sequencing data were collected from public databases including GEO, ENA, and SRA. (B) The RNA sequencing raw data were subjected to quality control, mapping, transcript quantification, and normalization to obtain high-quality data for further analysis. (C-D) Biofilm-associated networks and proteins were obtained via differential expression analysis, machine learning, prediction analysis, and WGCNA. (E) A comprehensive database called SAdb was developed.

**Fig. 2:**
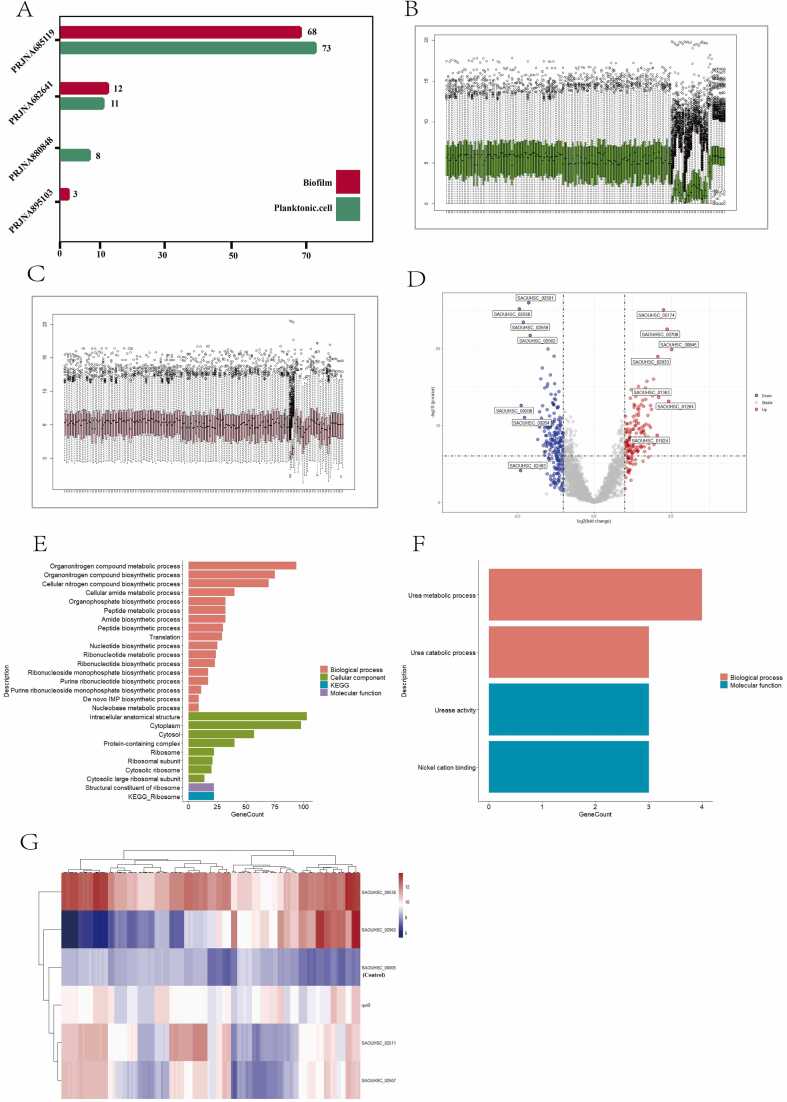
Acquisition of differentially expressed genes and corresponding GO and KEGG analysis. (A) Source and size of RNA sequencing data, which also provides the size of the experimental samples. (B) The deployment of the boxplot exhibits batch effects among different datasets, owing to the varying discrepancies introduced by differing experimental conditions across batches. (C) The employment of the sva combat function exterminates batch-related discrepancies, aiming to procure a dataset of elevated quality. The boxplot shows that the gene expression profiles of different samples were more consistent than those in Figure B, indicating the efficacy of the normalization method we employed. (D) Volcano plots were used to show differentially expressed genes that met the specified criteria (|Log2FC= >1, p-value < 0.01). (E) GO and KEGG analyses of DEGs to identify enriched functional pathways. (F) Selection of the top five upregulated and downregulated genes for subsequent GO and KEGG analyses to determine enrichment pathways. (G) Assessment of the utility of internal reference genes in *Staphylococcus aureus*. We used *gyrB* as a control to ensure the accuracy and reliability of the reference gene expression analysis in this study.

For the initial raw data collection, four ***Staphylococcus aureus*** transcriptome (RNA-seq) datasets in biofilm research were downloaded and curated, in a total of 175 samples (Fig. 2**A**), from the European Nucleotide Archive (EBI ENA, https://www.ebi.ac.uk/ena/browser/home) [12], and the Sequence Read Archive (SRA, https://www.ncbi.nlm.nih.gov/sra/) [13]. For detailed information on the datasets, 1) the first dataset GSE213381 is from Bertrand’s research (Role of *Staphylococcus aureus* formate metabolism during prosthetic joint infection) [14]. In this study, RNA sequencing was performed to evaluate how the *S. aureus* biofilm transcriptome was altered following exposure to a G-MDSCs or macrophages in vitro, with biofilm was used as control, totaling three replicates. Two of three samples met quality requirements and were grouped under biofilm conditions and used for subsequent research; 2) The second dataset, GSE216751, is from Yuki’s research (GltS regulates biofilm formation in methicillin-resistant *S. aureus*) [15]. In this study, *S. aureus* JE2 strain was cultured in vitro for biofilm formation, and samples were obtained at different time points for RNA sequencing; 3) The third dataset, PRJNA685119, is from Tomlinson’s research (A global transcriptomic analysis of *Staphylococcus aureus* biofilm formation across diverse clonal lineages) [16]. In this study, *S. aureus* biofilms were formed in TSB using 96-well plates with five *S. aureus* strains: N315 (USA100), MRSA252 (USA200), LAC (USA300), MW2 (USA400), and NRS385 (USA500). Biofilm samples and their planktonic counterparts were collected at 5 h, 10 h, and 24 h; 4) The fourth dataset PRJNA682641 is from Martin’s research (The de novo purine biosynthesis pathway is the only commonly regulated cellular pathway during biofilm formation in TSB-based medium in *Staphylococcus aureus* and *Enterococcus faecalis*) [17]. In this study, biofilm formation mRNA profiles of *S. aureus* SH1000 and *S. aureus* USA300 were compared to their planktonic state, in either TSBg(s) or BHIg(s) medium, in 3 biological replicates. Finally, 90 samples were obtained under biofilm conditions and 85 samples were obtained under planktonic conditions. For the processing of raw sequencing reads, FastQC (http://www.bioinformatics.babraham.ac.uk/projects/fastqc/) was used to evaluate the overall quality of the raw sequencing reads, followed by the Trim_galore to remove sequencing vectors and low-quality bases [18] and performed transcript quantification using Salmon [19], which adopted TPM (Transcript Per Million) for normalization, a better unit for RNA abundance than RPKM and FPKM since it respects the invariance property and is proportional to the average relative RNA molar concentration [20]. All RNA-seq samples were mapped to *Staphylococcus aureus* reference transcripts derived from the NCTC 8325 reference strain genome in the microbesonline database [21]. From four datasets, data was integrated on *Staphylococcus aureus* biofilm phenotype via the Sva R package Combat function and performed differential expressed analysis, in which a cutoff of |log2 FC= >1.0, (FC, fold change) and p-value < 0.05, to define DEGs (differentially expressed genes) between conditions were used. Subsequently, the DEGs obtained for the construction of machine learning models were utilized, and through the feature gene selection strategy, filtered DEGs were used to identify feature genes that made significant contributions to distinguishing the biofilm phenotype and planktonic state. Feature genes were analyzed using a previously developed biofilm-associated protein predictor [22]. Finally, the WGCNA pipeline (https://github.com/ShawnWx2019/WGCNA-shinyApp) was used to analyze the entire gene expression table and generate a co-expression network. The workflow of the analysis is illustrated in Fig. 1.

### 2.2 Data integrating and analysis

Differentially expressed genes in the RNA sequencing data were compared, as shown in Fig. 2**A**. Boxplot analysis, which is designed to reveal the central tendency and distribution of datasets, and t-SNE analysis, which is a widely employed dimensionality reduction technique are predominantly utilized for visualizing high-dimensional data and can accurately reflect the structure and distribution of the data in the lower-dimensional space (Fig. S1). Through boxplot and t-SNE analysis, a batch effect was found in the expression profile between different batches of items from RNA-seq (Fig. 2**B**) owing to the varying discrepancies introduced by the different experimental conditions across batches. The data were normalized using the Sva R package Combat function. The boxplot results showed that the gene expression profiles of the different samples were more consistent than those before (Fig. 2**C**), indicating the efficacy of the normalization method. These results indicated that the integrated data originating from different datasets can be used for further analysis. Next, the transcriptional profiles of *Staphylococcus aureus* samples were examined, and 409 DEGs were obtained through differential expression analysis (Fig. 2**D**), accounting for 14 % of the total number of genes, indicating that multiple pathways in *Staphylococcus aureus* are affected by the transition of bacteria from planktonic to biofilm state.

**Fig. 3:**
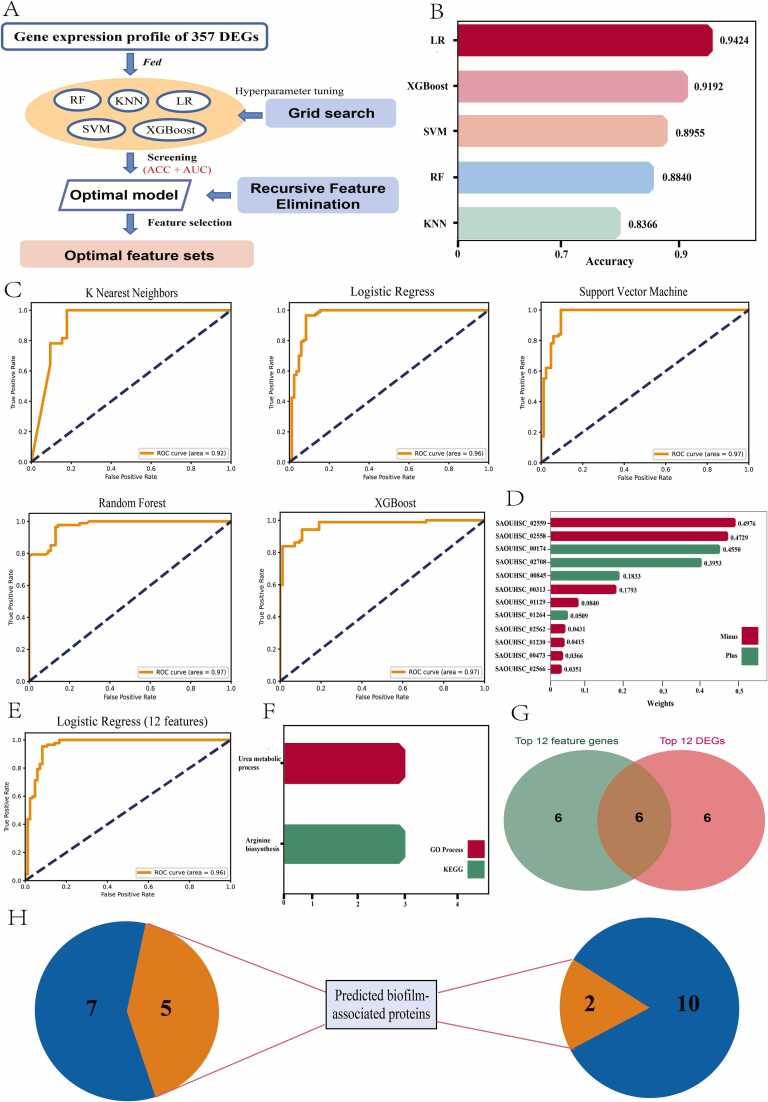
Using machine learning analysis to explore feature genes. Schematic of machine learning analysis. incorporating DEGs as data features for training machine learning models is presented, noting that genes encoding non-coding proteins within DEGs were filtered out to refine feature genes for model training. Grid search and RFE were utilized to determine the hyperparameters and optimal feature subsets, respectively. (B) Evaluation of performance among diverse machine learning algorithms including LR (Logistic Regression), XGBoost (eXtreme Gradient Boosting), SVM (Support Vector Machine), RF (Random Forest), KNN (K-Nearest Neighbors) through accuracy comparison. The results indicated that the model constructed using the LR algorithm exhibits optimal performance (accuracy). (C) Efficiency of different machine learning algorithms assessed by comparing the area under the ROC curve. The results indicated that models constructed using the SVM, RF, and XGBoost algorithms exhibited the best performance, achieving a ROC of 0.97. The model built with the LR algorithm attained a ROC value of 0.96, while KNN recorded the lowest at 0.92. (D) Computation of the contribution scores of feature genes to ascertain the optimal feature subset, culminating in a subset encompassing 12 feature genes including SAOUHSC_02559 (weight = −0.4976), SAOUHSC_02558 (weight = −0.4729), SAOUHSC_00174 (weight = 0.4550), SAOUHSC_02708 (weight = 0.3953), SAOUHSC_00845 (weight = 0.1833), SAOUHSC_00313 (weight = −0.1793), SAOUHSC_01129 (weight = −0.0840), SAOUHSC_01264 (weight = 0.0509), SAOUHSC_02562 (weight = −0.0431), SAOUHSC_01230 (weight = −0.0415), SAOUHSC_00473 (weight = −0.0366) and SAOUHSC_02566 (weight = −0.0351). (E) AUC value of the optimal feature subset in the LR model. The results indicated that the LR predictive model constructed on 12 feature genes performs equivalently to the model based on 357 DEGs (differentially expressed genes). (F) Pathway enrichment outcomes for a subset of 12 genes. The results indicated an enrichment in pathways germane to urea metabolism and arginine biosynthesis. (G) Overlapping analysis between features genes and Top DEGs. And 6 genes are shared by both sets, which indicated a degree of concordance in the conclusions derived through both methods. (H) Prediction of feature genes and Top DEGs using biofilm-associated protein predictors. A predictive tool (http://124.222.145.44/#!/score) was employed to analyze feature genes and Top DEGs in order to identify potential biofilm-associated proteins. The results indicated that feature genes (41.6 %) have a greater likelihood of being potential biofilm proteins compared to the top DEGs (16.7 %).

The subsequent functional enrichment, facilitated by the Gene Ontology (GO) [23] and Kyoto Encyclopedia of Genes and Genomes (KEGG) [24] databases, provided significant insights into biological functions. The GO analysis (Fig. 3B) primarily highlighted aspects such as biosynthesis, cytoplasm, and ribosomal structure, which are key indicators of growth, intracellular activities, and transcriptional mechanisms in *Staphylococcus aureus* (Fig. 2**E**). Similarly, the KEGG enrichment analysis identified ribosomes as the most critical pathway. Collectively, both GO and KEGG enrichment analysis revealed the rapid transcriptional and translational responses of bacterial genes and proteins to phenotypic alterations.

Further exploration was directed at the principal DEGs evident during the transition between the biofilm and planktonic states of the bacteria. The quintets of genes that were most notably downregulated at the whole transcriptome level included *SAOUHSC_02558* (*ureA*) and *SAOUHSC_02559* (*ureB*), which are associated with nitrogen metabolism and urea degradation, alongside *SAOUHSC_02493,* a 50S ribosomal protein implicated in *Staphylococcus aureus* translation, as identified in a previous study [25]. *SAOUHSC_00208* and *SAOUHSC_00264* were denoted as coding for hypothetical proteins. Additionally, prominent genes, such as *SAOUHSC_02561* (*ureC*) and *SAOUHSC_02562* (*ureE*), which are annotated as urease subunit alpha and urease accessory proteins, respectively, in the urea catabolic process within the KEGG pathway (Fig. 2**F**), but their functions in *Staphylococcus aureus* remain unknown.

Internal reference genes are important for transcription-level studies. According to literature [26], [27], [28], *gyrB* (*SAOUHSC_00005*) is stably expressed under different conditions and is considered a housekeeping gene as an internal reference gene. Therefore, *gyrB* gene was used as a control to ensure the accuracy and reliability of the reference gene expression analysis in this study. The internal reference genes selected for *Staphylococcus aureus* transcription are *tuf* (*SAOUHSC_00530*) [29], *rplD* (*SAOUHSC_02511*) [30], *rpoB* [31], *rplV* (*SAOUHSC_02507*) [32], and *SAOUHSC_02965*. The expression levels of *SAOUHSC_02965*, *SAOUHSC_02507*, and *rplD* were unstable under the different conditions (Fig. 2**G**). These genes exhibited varied expressions under different experimental conditions. Choosing these genes as internal reference genes was risky.

### 2.3 Using Machine learning analysis to explore feature genes

The utilization of machine learning in the domain of disease diagnostics encompasses a vast array of applications, ranging from the analysis of imagery to the intricacies of genomics, facilitating the early detection of diseases and forecasting the efficacy of treatments [33], [34]. Nonetheless, the exploration of bacterial phenotypes using machine learning methodologies remains relatively unexplored. In this study, machine learning algorithms were coupled with strategies for feature selection to identify crucial pathways that undergo modification as bacteria metamorphose from the planktonic to biofilm state, encapsulating processes such as oxidative respiration, metabolic activities, and biofilm formation. Initially, we employed expression data from 357 DEGs for the machine learning modeling datasets, as genes showing no significant changes in expression levels under two distinct experimental conditions (planktonic cell vs. biofilm) offered no substantial contribution to distinguishing the experimental conditions, rendering it impossible to infer the corresponding experimental conditions based solely on the gene expression levels of these genes. Furthermore, from a machine learning model construction perspective, the inclusion of genes without significant expression variation as features augments the complexity of the model, predisposing it to overfitting. Subsequent to establishing these data inputs, a comparative analysis was conducted on the model performance using algorithms such as Logistic Regression (LR), XGBoost, Support Vector Machines (SVM), K-Nearest Neighbors (KNN), and Random Forest (RF), leveraging ten-fold cross-validation and optimizing hyperparameters via grid search (Fig.3**A**). The findings revealed model accuracy spanning from 83 % to 94 % (Fig.3**B**), with Area under the Curve (AUC) scores ranging from 0.92 to 0.97 (Fig.3**C**). The model devised using the LR algorithm was distinguished by achieving an accuracy of 94.24 % and accompanied by an AUC score of 0.97, thereby selecting the LR algorithm for further feature selection and model refinement. Recursive Feature Elimination (RFE) is a feature selection procedure that iteratively reduces the number of features [35]. In the current study, an optimal subset of 12 genes was ascertained through application of the recursive feature elimination approach, and their contributions and significance to the model were meticulously evaluated (Fig. 3**D**). These genes are instrumental in the bacterial transition from planktonic to biofilm state. Ten-fold cross-validation ascertained that the LR model, when refined to include these 12 feature genes, achieved an accuracy of 93.07 %, with an AUC of 0.96 (Fig. 3**E**), which was nearly identical to that of the LR model constructed using the 357 DEGs. This highlights that the majority of DEGs did not play a pivotal role in the construction of the model. Gene Ontology (GO) and Kyoto Encyclopedia of Genes and Genomes (KEGG) analysis conducted on the 12 genes revealed an enrichment in pathways germane to urea metabolism and arginine biosynthesis (Fig. 3**F**). Remarkably, one-third of these genes lacked functional annotation. Moreover, by selecting DEGs based on log2 FC and p-values, the top 12 genes also showed enrichment in the urea metabolism pathway, reinforcing the pivotal role of urea metabolism in the bacterial transformation process. A subsequent overlapping analysis between the genes deemed as features and the top 12 DEGs uncovered 6 genes common to both sets, primarily enriched in the urea metabolism pathway, suggesting a degree of concordance in the conclusions derived through both methodologies (Fig.3**G**). Intriguingly, these genes were not enriched in pathways directly associated with biofilm development in the public databases. Therefore, the pathway datasets were delved for *Staphylococcus aureus* within the GO and KEGG databases, and the absence of annotations related to biofilm pathways was discovered. This suggests that the currently available gene annotation data for *Staphylococcus aureus* are less defined, thereby limiting our understanding of the biofilm formation pathways in *Staphylococcus aureus.*

To elucidate the linkage between these genes and the biofilm regulatory network, the proteins encoded by these genes were analyzed using a previously developed predictor of bacterial biofilm-associated proteins. The analysis identified that, among the 12 feature genes, five were predicted to be associated with biofilm formation, among which three were hypothetical proteins, such as *SAOUHSC_00313*. The gene *SAOUHSC_02562* (*ureE*), a urease accessory protein, is involved in the stability of biofilms in *Staphylococcus aureus* [36]. *SAOUHSC_02566* (*sarR*) is recognized as an HTH-type transcriptional regulator critical for biofilm formation by *Staphylococcus epidermidis* [37]. Among the 12 DEGs, the hypothetical protein *SAOUHSC_00208* and the Type VII secretion system extracellular protein *SAOUHSC_00264* were predicted to be potential biofilm-associated proteins (Fig.3**F**).

In this study, the method of feature gene selection via machine learning, compared to the method of Top DEGs, could enrich functional pathways that change during condition transitions while also uncovering potential biofilm-associated proteins with the predictor. However, in terms of numerical yield, the machine learning approach unravels a greater number of proteins, indicating that, in the context of handling expansive gene expression datasets, the strategy of feature gene selection via machine learning may prove more efficacious in unearthing important genes.

### 2.4 WGCNA elucidated the main functions of the modules

**Fig. 4:**
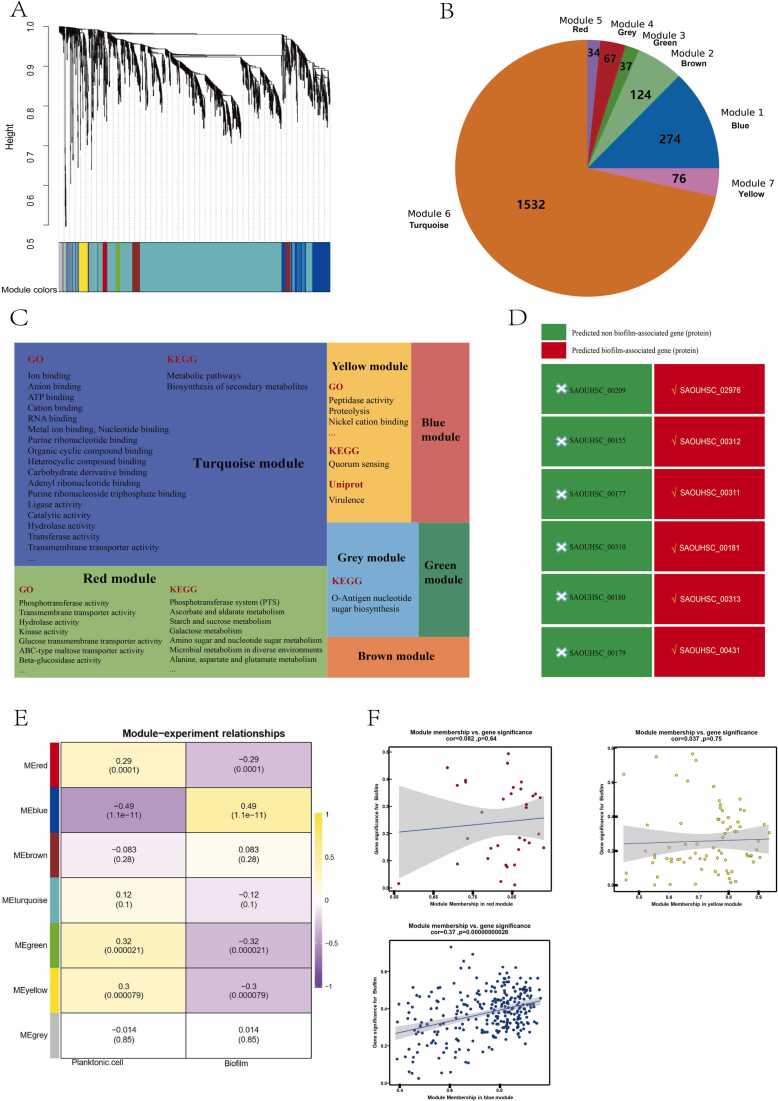
WGCNA elucidates the main functions of these modules. (A) Construction of a hierarchical clustering tree based on the distance matrix (TOM). (B) Size of gene modules within WGCNA and the respective gene members contained within these modules. In this study, we employed the dynamic tree cut method to prune the clustering tree and identified gene modules. (C) Dendrogram showing functional pathways enriched by gene modules. The results indicated Turquoise, Yellow, Red modules can be enriched in the pathways of the GO and KEGG databases. (D) Prediction of 12 feature genes of machine learning analysis using a biofilm-associated protein predictor, which is an online websever for prediction (http://124.222.145.44/#!/score). (E) Analysis of the association between gene modules and experimental conditions, including planktonic and biofilm states. The results indicated that biofilm phenotype was negatively correlated with the Red, Green, and Yellow modules, and positively correlated with the Blue module. (F) Correlation analysis of genes within the red, yellow, and blue modules in relation to the experimental conditions, which is used to analyze the significance of genes in relation to modules and biofilm phenotype.

WGCNA is a bioinformatics analysis method that reveals the interactions between gene modules and the associations between these modules and external phenotypic traits by constructing co-expression networks among genes [38]. In this study, the co-expressed gene network in *Staphylococcus aureus* transcripts was revealed by the WGCNA pipeline, which performed cluster analysis using hierarchical clustering (Fig. 4**A**), and distributed the filtered genes into 7 modules (Fig. 4**B**), with 274, 124, 37, 67, 34, 76, and 1532 genes in each module, respectively (Table 1).

**Table 1:** Gene modules obtained via WGCNA.

Modules	Genes	Unannotated genes	Non-protein coding genes
Turquoise	1532	766 (50.0 %)	3 (0.2 %)
Blue	274	193 (70.4 %)	3 (1.1 %)
Brown	124	105 (84.7 %)	0 (0 %)
Yellow	76	41 (53.9 %)	0 (0 %)
Grey	67	42 (62.7 %)	0 (0 %)
Green	37	8 (21.6 %)	20 (54.1 %)
Red	34	15 (44.1 %)	0 (0 %)

The Turquoise module contains 1532 gene members, of which 766 (50 %) are currently unannotated. Functional enrichment analysis of the Turquoise module mainly focused on the cellular metabolism, biosynthesis, and membrane transport pathways (Fig. 4**C**). The Yellow module is associated with bacterial virulence and quorum sensing (Fig. 4**C**). The enrichment of the Grey module predominantly revolves around the O-antigen nucleotide sugar biosynthesis pathways (Fig. 4**C**). The Red module was significantly enriched in the phosphotransferase system (PTS), ascorbate and aldarate metabolism, and amino sugar and nucleotide sugar metabolism (Fig. 4**C**). The Blue, Brown, and Green modules were not enriched in specific functional pathways (Fig. 4**C**), which may be attributed to the high presence of unannotated or non-protein coding genes, with unannotated gene counts of 196 (71.5 %), 105 (84.7 %), and 28 (75.7 %), respectively. The main objectives of this study were to identify gene modules closely related to the biofilm phenotype by analyzing the association between experimental conditions (biofilm and planktonic conditions) and gene modules. The results showed that the biofilm phenotype was negatively correlated with the Red (−0.29, 0.0001), Green (−0.32, 0.000021), and Yellow (−0.3, 0.000079) modules, and positively correlated with the Blue module (0.49, 1.1e-11), indicating that when bacteria transition from a planktonic state to a biofilm state, their virulence regulation system and energy metabolism pathways, including vitamins, sugar acids, and urea metabolism, are affected (Fig. 4**E**). Notably, 54.1 % of the gene members in the Green module encode tRNA and rRNA, with 21.6 % remaining uncharted, leading to discontinuation in the exploration of its functionalities. Subsequently, hub gene screening analysis of the Red, Yellow, and Blue modules were conducted to enhance understanding of the their functionality expressed by these modules (Fig. 4**F**). The hub genes of the three modules were obtained by screening with |gene significance (GS)| > 0.2 and |module membership (MM)| > 0.8, and were analyzed for functional enrichment again. The results showed that In the Red module, 4 hub genes were enriched in ascorbate and aldarate metabolism pathways, 6 hub genes in the PTS system, and 4 hub genes in amino sugar and nucleotide sugar metabolism. For unidentified gene members within this module, such as SAOUHSC_00313, the strategy of analyzing unknown genes within the same module via hub genes located in known pathways proved valuable. Moreover, research has shown that the phosphotransferase system (PTS) is involved in biofilm formation [39], [40]. Analysis of hub genes in this module using a predictor revealed that 6 (50 %) genes encoded biofilm-associated proteins, suggesting that this module may be correlated with the biofilm regulatory pathway (Fig. 4**D**). In the Yellow module, four (33.4 %) hub genes were enriched in the Type VII secretion system (T7SS) functional pathway, which is known to secrete pathogenic factors that facilitate bacterial invasion of host cells and disrupt their normal functions, thus augmenting the survival and proliferation of pathogens [41]. Furthermore, T7SS aids bacteria in resource competition with other microbes [42]. Proteins secreted by T7SS can directly or indirectly curtail the growth of competitors, thereby enhancing bacterial competitiveness and survival. Hub gene analysis of the Blue module, which showed the strongest association with the biofilm phenotype, revealed that his module was mainly enriched in the stress response, chaperone, and other functional pathways. Additionally, the literature suggests that multiple hub genes are involved in biofilm formation, virulence regulation, and bacterial pathogenicity [43], [44].

### 2.5 PPI interaction networks between hub genes suggest novel interactions

**Fig. 5:**
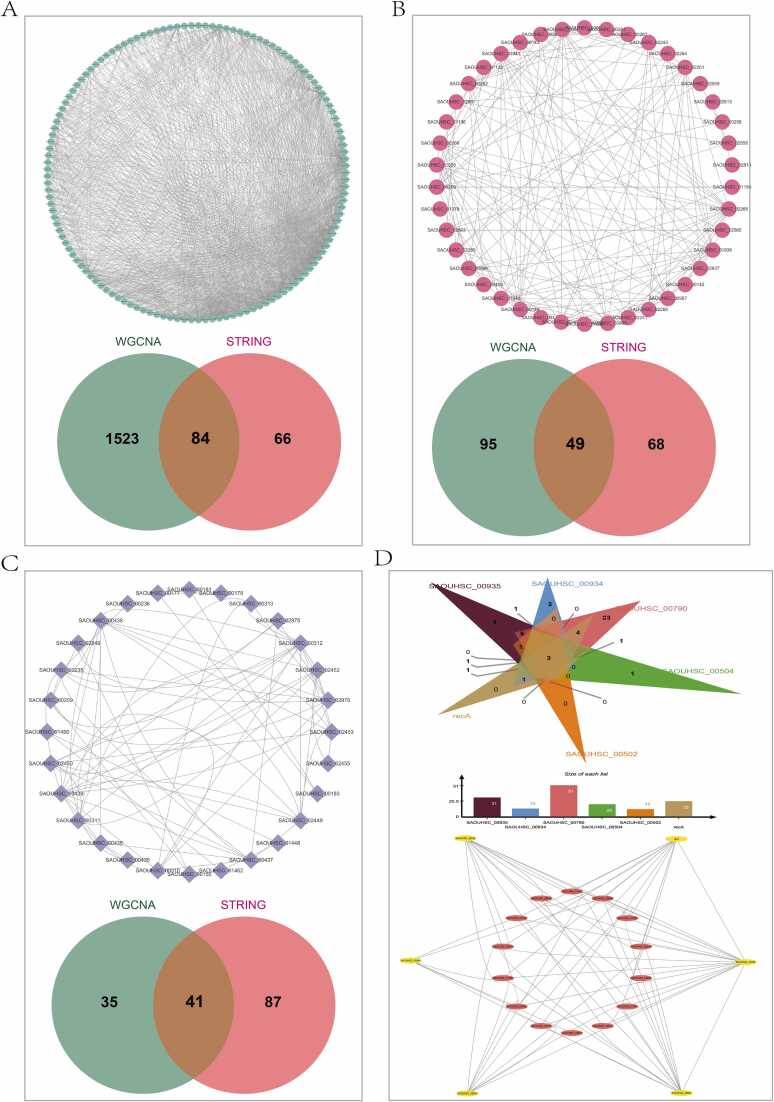
PPI interaction networks between hub genes, suggesting novel interactions. The interaction analysis of hub genes within the blue module and the overlapping analysis of interactions were obtained using the STRING database and WGCNA. (B) Interaction analysis of yellow module hub genes with an overlapping analysis of interactions derived from both the STRING database and WGCNA. (C) Interaction analysis of hub genes within the red module and overlapping analysis of interactions obtained through the STRING database and WGCNA. (D) Elucidation of six experimentally validated genes encoding biofilm-associated proteins and their respective PPI networks, with overlapping analysis to discern shared genes and potentially unveil a subnetwork within the biofilm formation pathway.

Gene co-expression usually implies interactions or regulatory relationships among shared genes. Thus, gene co-expression data obtained from the WGCNA analysis were used to construct the interaction networks. To compare with existing interaction data in public databases, these genes were entered into the STRING database, a collection of known and predicted direct physical binding and indirect functionally related interactions between proteins and genes, and obtained their Protein-Protein Interaction (PPI) networks were obtained [45]. First, PPI network mapping of hub genes was performed within the three modules using gene co-expression relationships obtained from WGCNA by setting a weight threshold of the top 10 %. The PPI networks constructed for hub genes of the yellow and red modules demonstrated tight interactions, and functional enrichment was consistent with previous GO/KEGG analysis. Interestingly, the interaction of hub genes from the Blue module sourced from the STRING database was significantly lower than that from WGCNA. Moreover, the yellow module was found to have a higher proportion of PPIs shared between both sources, accounting for 34 % of the WGCNA and 41.9 % of the STRING sources (Fig. 5**A**). In the red module, the shared PPIs from the WGCNA and STRING sources accounted for 54 % and 32 %, respectively (Fig. 5**B**). The blue module had the least shared PPIs, accounting for 5.2 % of WGCNA and 56 % of STRING sources (Fig. 5**C**). The variability of co-expression relationships in the blue module, as indicated by STRING data, suggests that there may be many new protein interactions within this module. Considering that the blue module had the strongest association with the biofilm phenotype, members of the biofilm regulatory network might be located within this module, which are genes that are yet to be explored. Using literature and public databases, we identified 6 genes encoding biofilm-associated proteins (Fig. 4**D**), and was validated using a biofilm-associated protein predictor. Based on gene co-expression data from WGCNA, PPI networks were obtained for every predicted biofilm-associated gene, in total obtaining 6 networks. By comparing the gene members of each network and conducting overlapping analysis, 3 genes shared by all 6 networks, 7 shared by 5 networks, and 4 shared by 4 networks were identified. The results suggest that these 14 genes, which are shared by most networks, are highly likely to be related to the biofilm regulatory network. Then, the connections of these 14 shared genes with the 6 biofilm-associated genes from WGCNA were extracted to construct a PPI network, which might represent a subnetwork of the biofilm regulatory network (Fig. 5**D**). However, these genes and their interactions require further validation.

### 2.6 The development of a comprehensive database of *Staphylococcus aureus*

**Fig. 6:**
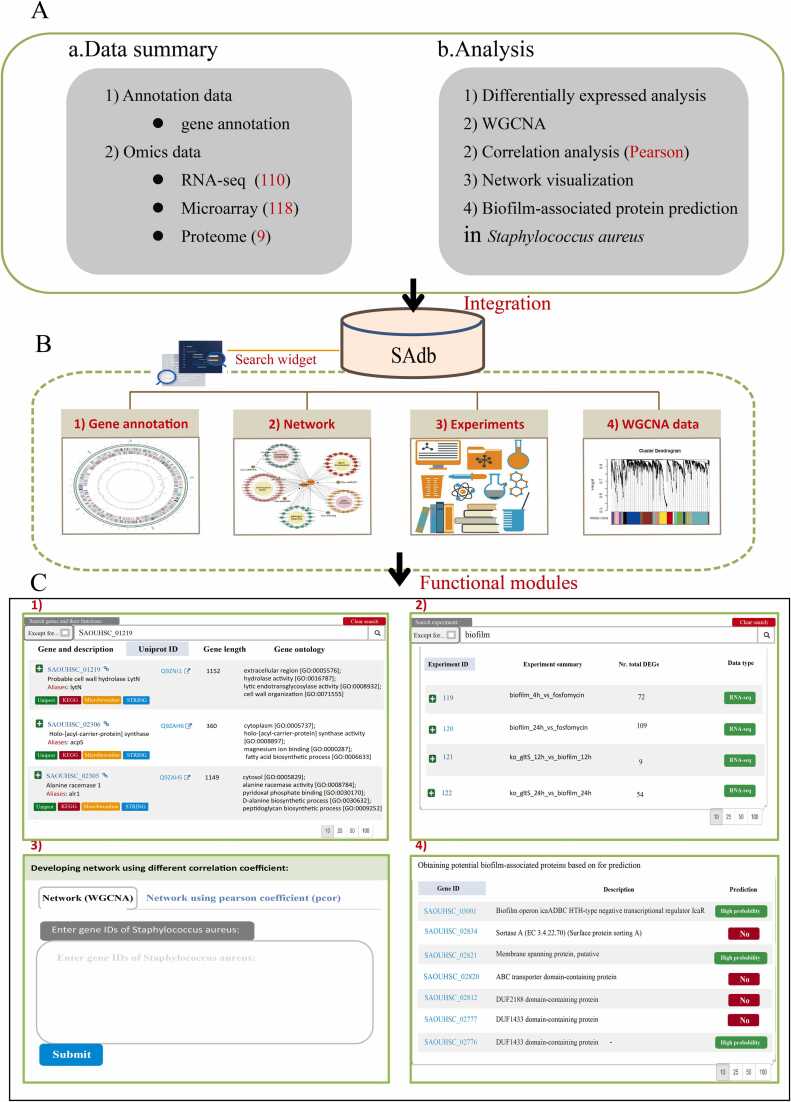
Overview of SAdb. (A) SAdb furnishing omics data along with the corresponding analysis functionalities. (B) SAdb database includes “Genes”, “Network”, “Experiments”, and “Results”. (C) Each functional module in the SAdb is visually demonstrated.

The significance of making research data readily accessible cannot be overlooked. To facilitate expeditious access to the data encompassed in this study for scholars in the field, a database named SAdb was established (Fig. 6). Concurrently, comprehensive gene annotation information for *Staphylococcus aureus*, as well as transcriptomic data, including both RNA-seq and microarray, along with proteomic data (Fig. 6**A**). Utilizing this comprehensive suite of data, differential expression analysis was performed to obtain differentially expressed proteins, and gene and gene correlation analysis were conducted to obtain the correlations between genes, which were subsequently depicted via network visualization. These data and subsequent analyses constituted four pivotal functional modules of the SAdb database (Fig. 6**B**). The “Gene” module is used to display gene annotation information on *Staphylococcus aureus*. The “Experiments” module collects experimental information on transcriptomics and proteomics. Clicking on a particular “Nr. Total DEGs” entry will provide detailed information on the DEGs under the corresponding experiment. In “Network” module, users can obtain the co-expression network of genes of interest through the search functionality. The “Results” module is used to display the data generated through this study (Fig. 6**C**). Hope that this database can serve as infrastructure for the biofilm research community.

## 3 Discussion

The exploration of biofilm formation by *Staphylococcus aureus* represents a pivotal area of inquiry within the domains of medical and biological research. Elucidating the underpinnings of biofilm development is instrumental in forging innovative therapeutic modalities aimed at dismantling bulwarks of bacterial defiance, thereby providing novel methodologies for the amelioration of associated infections. In this study, transcriptomic data from four experiments on *Staphylococcus aureus* biofilm phenotype, totaling 175 samples, were integrated. Given that these samples originated from four different experimental conditions, it is imperative to standardize them to eliminate batch effects to the greatest extent possible. Furthermore, other samples under the four experimental conditions, such as the biofilm condition with exposure to macrophages or the biofilm condition with gene inactivation (*gltS*), were excluded because they involved additional influencing factors. Through comprehensive analysis, hope to identify the bacterial molecular pathways that affect the bacterial transition from the planktonic to biofilm state, as well as the regulatory networks of biofilm formation.

Differential expression analysis was performed to obtain 409 differentially expressed genes (DEGs). Interestingly, the GO and KEGG enrichment results revealed that only 32 % (131/409) of the DEGs were enriched, whereas the remaining 68 % did not enrich any molecular pathways. This indicates that the annotation of *Staphylococcus aureus* using public databases, including genes and functional pathways, is not comprehensive, which greatly increases the analytical challenge. A differential fold ranking method was used to identify the top 5 upregulated and the top 5 downregulated genes. GO and KEGG analysis results showed that the urea metabolism pathway was affected, consistent with the enrichment results of the feature genes selected using machine learning. The bacterial urea metabolism pathway, which is significant for environmental adaptability, virulence, and nitrogen utilization, indicates that during the transition from motile to biofilm state, pathogenicity is indeed affected, corroborating the postulation that “the infective process in the biofilm state is exponentially magnified in comparison to the motile state” [46].

The utilization of a differential fold ranking method, while rudimentary, overlooks certain genes with pivotal roles yet minute variations in expression, thus diverting from the research focus [47]. GSEA has emerged as a methodology concentrating on whether a pre-ordered gene assembly exhibits systemic differential expression, providing a panoramic view of biological processes, pathways, and functionalities [48]. Despite the inconsequential alterations in the expression of solitary genes, the collective gene set may still have significant biological relevance. Nevertheless, the limited functional annotation information for *Staphylococcus aureus* limits the availability of gene set data required for GSEA analysis. The application of machine learning analysis, the consideration of the variations in gene expression under different experimental conditions and the consistency of under the same experimental conditions, to gene expression data often identifies important genes related to phenotypes. Therefore, machine learning analysis was used to screen for feature genes and obtained a total of 12. In contrast to the Top 12 genes, these feature genes were enriched in more functional pathways, suggesting that machine learning analysis can capture a more comprehensive picture of pathway alterations. Additionally, several genes among the feature genes were predicted to encode biofilm-associated proteins, consistent with previous literature [49], whereas fewer genes encoding biofilm-associated proteins were predicted among the Top genes. Comparing these two methods, sufficient samples of gene expression data was found that machine learning analysis may be more conducive to obtaining biologically meaningful genes than Top DEGs.

Gene network analysis, as opposed to single-gene analysis, can reveal the complexity and internal interactions of the system more comprehensively. Therefore, in this study WGCNA was used to obtain biologically meaningful gene modules. After obtaining the gene modules, hub gene analysis was performed on the three modules based on their relevance to the experimental conditions and module membership composition. Functionally, these hub genes are often enriched in pathways associated with bacterial biofilm formation, virulence regulation, and pathogenicity, demonstrating the reliability of the weighted gene co-expression network analysis in this study. In terms of the number of gene pairs, WGCNA obtained far more connections between genes than those currently recorded in the public databases. On the one hand, this shows that the construction of a protein-protein interaction network based on hub genes has discovered novel interactions, a desired outcome. However, given that weighted gene co-expression network analysis fundamentally hinges on Pearson’s correlation to construct gene associations, it cannot circumvent the influence of indirect gene relationships, inevitably leading to numerous gene pairs with elevated correlations that are devoid of biological import. Using direct gene relationships to construct a co-expression network epitomizes an efficacious strategy for harvesting high-caliber gene pairs.

To facilitate the reuse and analysis of the data in this study by researchers in the field and concurrently archive data pertinent to *Staphylococcus aureus*, a comprehensive database of *Staphylococcus aureus* was created. This database includes data generated from this study as well as published transcriptomic and proteomic data. Hopefully, this database will help researchers in the field better understand and analyze the genetic characteristics and pathogenesis of *Staphylococcus aureus*.

In summary, integrating machine learning with WGCNA to analyze bacterial biofilm pathways and their associated proteins can help researchers better understand the mechanisms of biofilm development and identify new potential biofilm-associated genes (proteins) in *Staphylococcus aureus*. For example, the previously mentioned gene of unknown function, *SAOUHSC_00313*, was predicted to be a biofilm-associated protein. Through sequence alignment, the homolog of *SAOUHSC_00313,* BglG, was identified, which are associated with virulence and biofilm formation in *Enterococcus faecalis* [50]. Furthermore, genes experimentally validated to be involved in the biofilm phenotype include *SAOUHSC_02562* (*ureE*), which is implicated in the stability of *Staphylococcus aureus* biofilms [36]. Additionally, literature mining revealed that several genes within the Blue module (over 70 % genes were unannotated) of a highly biofilm-phenotype-associated module identified in WGCNA, such as *SAOUHSC_00790* (*clpP*) [51], *SAOUHSC_01684* (*grpE*) [52], and *SAOUHSC_00812* (*clfA*) [53], have already been confirmed to regulate bacterial biofilm formation. However, this analytical approach has several limitations. 1) A series of analysis are predominantly based on transcriptomic data, neglecting other layers such as proteomic data; 2) in practical applications, disparities between different biofilm model systems, as well as numerous other factors, such as experimental duration and specific strains, must be considered to obtain condition-dependent biofilm-associated proteins [54], [55], which can effectively elucidate the diversity in bacterial biofilm development mechanisms; 3) While WGCNA is a highly esteemed method for constructing gene networks, it fails to exclude indirect interactions between genes, and employing alternative methods such as Gaussian graphical models (GGM) [56] to construct gene networks may prove to be more insightful.

## 4 Materials and methods

### 4.1 Data collection, pre-processing and analysis

The raw sequencing data were downloaded from ENA and SRA, which came from four experiments on the *Staphylococcus aureus* biofilm phenotype (GSE213381, PRJNA685119, PRJNA682641, and GSE216751), totaling 175 samples (Table S1). Evaluated the overall quality of raw sequencing reads using FastQC, removed sequencing adapters and low-quality bases with Trim_galore, and performed transcript quantification using Salmon. After obtaining the processed gene expression data, these data were integrated using the Combat function of the Sva R package, and differential expression analysis was performed to define differentially expressed genes between conditions with cutoff values |log2 FC= > 1.0 (FC, fold change) and p-value < 0.05. Additionally, the utility of the internal reference genes was assessed and the functions of the Top DEGs were analyzed.

### 4.2 Machine learning analysis

In this study, five popular ML algorithms, LR, XGBoost, SVM, KNN, and RF, were used to analyze the gene expression data to identify feature genes. During model construction, 357 differentially expressed genes (DEGs) were used as feature genes for model development. The expression data of 357 feature genes were extracted from 172 samples to serve as the data input for training the model (Table S2). Then, by comparing the performance (Accuracy and ROC) of the models constructed based on five different machine learning algorithms, the optimal algorithm for model construction was determined. Subsequently, a Grid search was employed to identify the optimal feature subset. Ultimately, a model is constructed using an optimal feature subset to reduce the number of features and the complexity of the model. All ML algorithms were implemented in Python and used a grid search and ten-fold cross-validation for hyperparameter optimization (Table S3).

### 4.3 WGCNA

Gene expression matrices were used to perform weighted gene co-expression network analysis using WGCNA (https://github.com/ShawnWx2019/WGCNA-shinyApp). This process included calculating the Pearson correlation matrix, adjacency matrix with power β = 3, and the final topological overlap matrix (TOM) based on normalized gene expression counts. Then, this TOM was filtered to exclude any samples containing weighted co-expressed < 0.1 in all analysis.

### 4.4 Online predictor

In this study, the BBSdb predictor, which were developed in previous study, was used to predict the Top DEGs, feature genes from the machine learning model, and hub genes from important modules obtained through WGCNA, to discover potential biofilm-associated proteins.

### 4.5 Database design and implementation

SAdb is a relational database in which all the data are loaded into the MySQL database. The website’s frontend is coded using JavaScript and HTML, while the backend is coded in PHP to support queries to the MySQL database and provide a Representational State Transfer (REST) application programming interface (API) for programmable access to available data. The AngularJS framework was used to connect the frontend and the backend. Echarts.js and plotly.js were used for front-end visualizations.

## CRediT authorship contribution statement

**Erguang Li:** Project administration, Methodology, Investigation, Funding acquisition, Conceptualization. **Yin Liu:** Visualization, Software. **Zhu Yang:** Visualization, Data curation. **Yuanyuan Pan:** Software, Resources, Investigation. **Wajid Hussain:** Writing – review & editing, Formal analysis. **Guozhong Chen:** Validation, Resources, Formal analysis, Data curation. **zhiyuan Zhang:** Writing – original draft, Visualization, Validation, Software, Resources, Methodology, Investigation, Formal analysis.

## Declaration of Competing Interest

The authors declare no conflicts of interest.

## References

[1] Cheung G.Y.C., Bae J.S., Otto M. (2021). Pathogenicity and virulence of *Staphylococcus aureus*. Virulence.

[2] Yan J., Bassler B.L. (2019). Surviving as a community: antibiotic tolerance and persistence in bacterial biofilms. Cell Host Microbe.

[3] Hall-Stoodley L., Costerton J.W., Stoodley P. (2004). Bacterial biofilms: from the natural environment to infectious diseases. Nat Rev Microbiol.

[4] Liu Z., Wang H., Zhou Z. (2016). Differential thiol-based switches jump-start *Vibrio cholerae* pathogenesis. Cell Rep.

[5] Wang S., Zhao Y., Breslawec A.P. (2023). Strategy to combat biofilms: a focus on biofilm dispersal enzymes. NPJ Biofilms Micro.

[6] Kierek K., Watnick P.I. (2003). Environmental determinants of *Vibrio cholerae* biofilm development. Appl Environ Microbiol.

[7] Poudel S., Hefner Y., Szubin R. (2022). Coordination of CcpA and CodY regulators in *Staphylococcus aureus USA300* strains. mSystems.

[8] Shibamura-Fujiogi M., Wang X., Maisat W. (2022). GltS regulates biofilm formation in methicillin-resistant *Staphylococcus aureus*. Commun Biol.

[9] Harkins C.P., Pichon B., Doumith M. (2017). Methicillin-resistant *Staphylococcus aureus* emerged long before the introduction of methicillin into clinical practice. Genome Biol.

[10] Langfelder P., Horvath S. (2008). WGCNA: an R package for weighted correlation network analysis. BMC Bioinforma.

[11] Swanson K., Wu E., Zhang A., Alizadeh A.A., Zou J. (2023). From patterns to patients: advances in clinical machine learning for cancer diagnosis, prognosis, and treatment. Cell.

[12] Li W., Cowley A., Uludag M. (2015). The EMBL-EBI bioinformatics web and programmatic tools framework. Nucleic Acids Res.

[13] Katz K., Shutov O., Lapoint R., Kimelman M., Brister J.R., O'Sullivan C. (2022). The sequence read archive: a decade more of explosive growth. Nucleic Acids Res.

[14] Bertrand B.P., Heim C.E., West S.C. (2022). Role of Staphylococcus aureus formate metabolism during prosthetic joint infection. Infect Immun.

[15] Shibamura-Fujiogi M., Wang X., Maisat W. (2022). GltS regulates biofilm formation in methicillin-resistant Staphylococcus aureus. Commun Biol.

[16] Tomlinson B.R., Malof M.E., Shaw L.N. (2021). A global transcriptomic analysis of Staphylococcus aureus biofilm formation across diverse clonal lineages. Micro Genom.

[17] Gélinas M., Museau L., Milot A., Beauregard P.B. (2021). The de novo Purine Biosynthesis pathway is the only commonly regulated cellular pathway during biofilm formation in TSB-Based Medium in Staphylococcus aureus and Enterococcus faecalis. Microbiol Spectr.

[18] Ozsolak F., Milos P.M. (2011). RNA sequencing: advances, challenges and opportunities. Nat Rev Genet.

[19] Patro R., Duggal G., Love M.I., Irizarry R.A., Kingsford C. (2017). Salmon provides fast and bias-aware quantification of transcript expression. Nat Methods.

[20] Zhao S., Ye Z., Stanton R. (2020). Misuse of RPKM or TPM normalization when comparing across samples and sequencing protocols. RNA.

[21] Dehal P.S., Joachimiak M.P., Price M.N. (2010). MicrobesOnline: an integrated portal for comparative and functional genomics. Nucleic Acids Res.

[22] Zhang Z., Pan Y., Hussain W., Chen G., Li E. (2024). BBSdb, an open resource for bacterial biofilm-associated proteins. Front Cell Infect Microbiol.

[23] (2019). The Gene Ontology Consortium. The gene ontology resource: 20 years and still GOing strong. Nucleic Acids Res.

[24] Kanehisa M. (2000). Goto S. KEGG: kyoto encyclopedia of genes and genomes. Nucleic Acids Res.

[25] Eyal Z., Matzov D., Krupkin M. (2015). Structural insights into species-specific features of the ribosome from the pathogen Staphylococcus aureus. Proc Natl Acad Sci USA.

[26] Margerrison E.E., Hopewell R., Fisher L.M. (1992). Nucleotide sequence of the Staphylococcus aureus gyrB-gyrA locus encoding the DNA gyrase A and B proteins. J Bacteriol.

[27] Azam M.A., Thathan J., Jubie S. (2015). Dual targeting DNA gyrase B (GyrB) and topoisomerse IV (ParE) inhibitors: a review. Bioorg Chem.

[28] Abass N.A., Suleiman K.M., El Jalii I.M. (2010). Differentiation of clinical Mycobacterium tuberculosis complex isolates by their GyrB polymorphism. Indian J Med Microbiol.

[29] Han H.W., Chang H.C., Chang T.C. (2016). Identification of Staphylococcus spp. and detection of mecA by an oligonucleotide array. Diagn Microbiol Infect Dis.

[30] Zanfardino A., Di Napoli M., Migliore F. (2023). Characterization of Linezolid-Analogue L3-Resistance Mutation in Staphylococcus aureus. Microorganisms.

[31] Aboshkiwa M., Rowland G., Coleman G. (1995). Nucleotide sequence of the Staphylococcus aureus RNA polymerase rpoB gene and comparison of its predicted amino acid sequence with those of other bacteria. Biochim Biophys Acta.

[32] Prunier A.L., Malbruny B., Laurans M., Brouard J., Duhamel J.F., Leclercq R. (2003). High rate of macrolide resistance in Staphylococcus aureus strains from patients with cystic fibrosis reveals high proportions of hypermutable strains. J Infect Dis.

[33] Hosny A., Parmar C., Quackenbush J., Schwartz L.H., Aerts H.J.W.L. (2018). Artificial intelligence in radiology. Nat Rev Cancer.

[34] Hamamoto R., Takasawa K., Shinkai N. (2023). Analysis of super-enhancer using machine learning and its application to medical biology. Brief Bioinform.

[35] Ravishankar H., Madhavan R., Mullick R., Shetty T., Marinelli L., Joel S.E. (2016). Recursive feature elimination for biomarker discovery in resting-state functional connectivity. Annu Int Conf IEEE Eng Med Biol Soc.

[36] Siddiqui H., Atia-Tul-Wahab, Ahmed A., Choudhary M.I. (2023). Structural and functional analysis of urease accessory protein E from vancomycin-resistance Staphylococcus aureus MU50 strain. Protein Pept Lett.

[37] Rowe S.E., Campbell C., Lowry C. (2016). AraC-type regulator Rbf controls the Staphylococcus epidermidis biofilm phenotype by negatively regulating the icaADBC repressor SarR. J Bacteriol.

[38] DuPai C.D., Wilke C.O., Davies B.W. (2020). A comprehensive coexpression network analysis in vibrio cholerae. mSystems.

[39] Horng Y.T., Wang C.J., Chung W.T., Chao H.J., Chen Y.Y., Soo P.C. (2018). Phosphoenolpyruvate phosphotransferase system components positively regulate *Klebsiella* biofilm formation. J Microbiol Immunol Infect.

[40] Houot L., Chang S., Pickering B.S., Absalon C., Watnick P.I. (2010). The phosphoenolpyruvate phosphotransferase system regulates *Vibrio cholerae* biofilm formation through multiple independent pathways. J Bacteriol.

[41] Kengmo Tchoupa A., Watkins K.E., Jones R.A. (2020). The type VII secretion system protects Staphylococcus aureus against antimicrobial host fatty acids. Sci Rep.

[42] Spencer B.L., Job A.M., Robertson C.M. (2023). Heterogeneity of the group B streptococcal type VII secretion system and influence on colonization of the female genital tract. Mol Microbiol.

[43] Mietrach N., Damián-Aparicio D., Mielich-Süss B., Lopez D., Geibel S. (2020). Substrate Interaction with the EssC coupling protein of the type VIIb secretion system. J Bacteriol.

[44] Battesti A., Gottesman S. (2013). Roles of adaptor proteins in regulation of bacterial proteolysis. Curr Opin Microbiol.

[45] Szklarczyk D., Kirsch R., Koutrouli M. (2023). The STRING database in 2023: protein-protein association networks and functional enrichment analyses for any sequenced genome of interest. Nucleic Acids Res.

[46] Moormeier D.E., Bayles K.W. (2017). Staphylococcus aureus biofilm: a complex developmental organism. Mol Microbiol.

[47] Zhang Z., Chen G., Hussain W. (2022). Mr.Vc v2: An updated version of database with increased data of transcriptome and experimental validated interactions. Front Microbiol.

[48] Oprescu S.N., Horzmann K.A., Yue F., Freeman J.L., Kuang S. (2018). Microarray, IPA and GSEA analysis in mice models. Bio Protoc.

[49] Zheng J., Wu Y., Lin Z. (2020). ClpP participates in stress tolerance, biofilm formation, antimicrobial tolerance, and virulence of Enterococcus faecalis. BMC Microbiol.

[50] Soussan D., Salze M., Ledormand P. (2023). The NagY regulator: A member of the BglG/SacY antiterminator family conserved in Enterococcus faecalis and involved in virulence. Front Microbiol.

[51] Liu Q., Wang X., Qin J. (2017). The ATP-dependent protease ClpP inhibits biofilm formation by regulating agr and cell wall hydrolase Sle1 in Staphylococcus aureus. Front Cell Infect Microbiol.

[52] Wang B., Song C.R., Zhang Q.Y. (2022). The fusaric acid derivative qy17 inhibits Staphylococcus haemolyticus by disrupting biofilm formation and the stress response via altered gene expression. Front Microbiol.

[53] Chen Q., Xie S., Lou X. (2020). Biofilm formation and prevalence of adhesion genes among Staphylococcus aureus isolates from different food sources. Microbiologyopen.

[54] Edel M., Horn H., Gescher J. (2019). Biofilm systems as tools in biotechnological production. Appl Microbiol Biotechnol.

[55] Flemming H.C., van Hullebusch E.D., Neu T.R. (2023). The biofilm matrix: multitasking in a shared space. Nat Rev Microbiol.

[56] Shutta K.H., De Vito R., Scholtens D.M., Balasubramanian R. (2022). Gaussian graphical models with applications to omics analyses. Stat Med.

