# Supplementary material for: Machine learning and network analysis with focus on the biofilm in Staphylococcus aureus
Source: Comput Struct Biotechnol J. 2024 Nov 10;23:4148–60. doi: 10.1016/j.csbj.2024.11.011 (PMC11617897; doi:10.1016/j.csbj.2024.11.011)
Supplement: Supplementary file 1 — Supplementary material [file mmc1.docx]

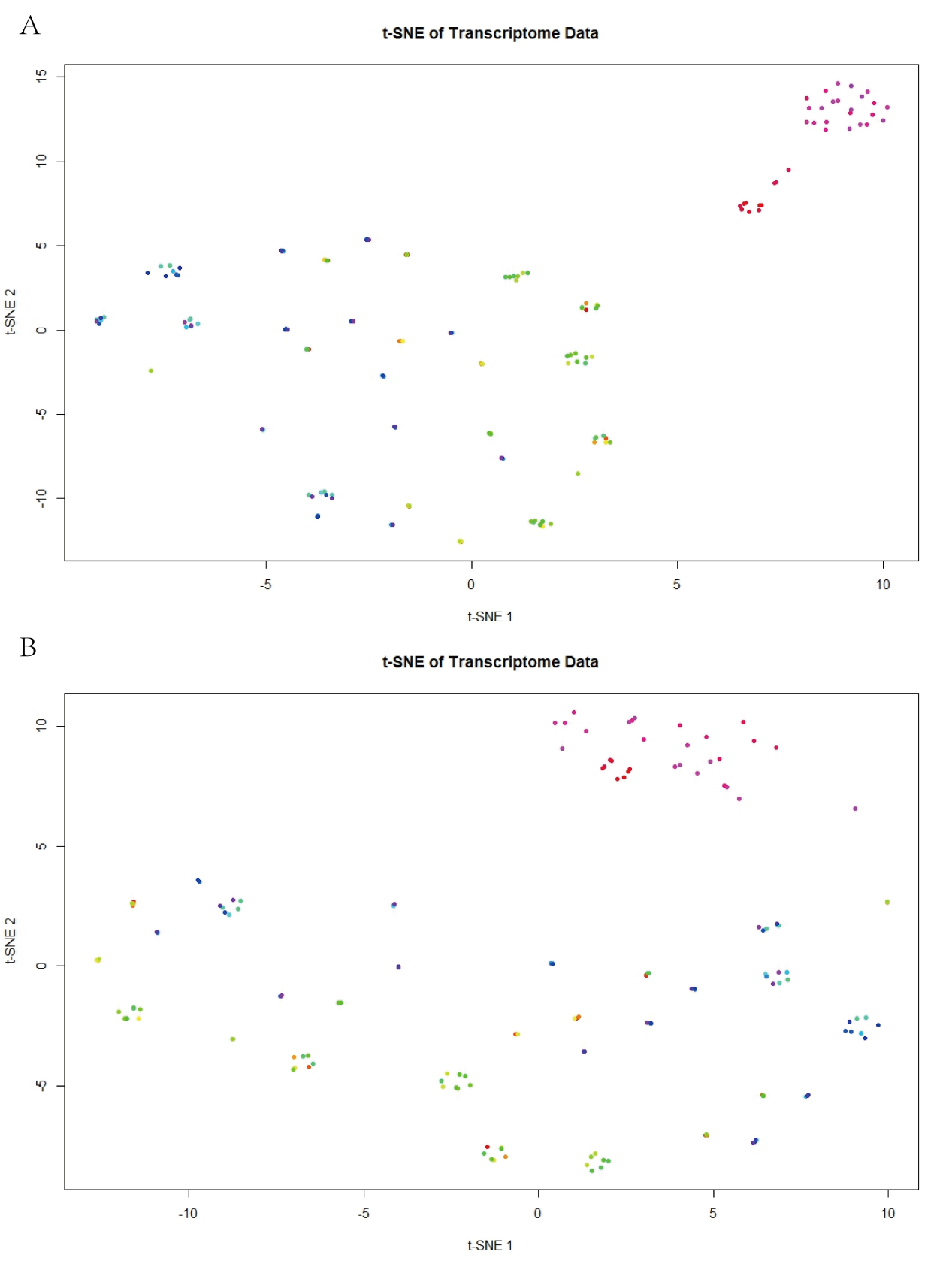


**Fig. S1** The deployment of box t-SNE analysis to exhibit batch effects among different datasets. (A) Spatial distribution of all samples before integration by R t-SNE dimensionality reduction. (B) Spatial distribution of all samples after integration by R t-SNE dimensionality reduction.
